# Worenine reverses the Warburg effect and inhibits colon cancer cell growth by negatively regulating HIF-1α

**DOI:** 10.1186/s11658-021-00263-y

**Published:** 2021-05-18

**Authors:** Lijiang Ji, Weixing Shen, Feng Zhang, Jie Qian, Jie Jiang, Liping Weng, Jiani Tan, Liu Li, Yugen Chen, Haibo Cheng, Dongdong Sun

**Affiliations:** 1 Changshu TCM Hospital Affiliated to Nanjing University of Chinese Medicine, Changshu, 215500 China; 2 Collaborative Innovation Center of Jiangsu Province of Cancer Prevention and Treatment of Chinese Medicine, Nanjing, 210023 China; 3 School of Integrated Chinese and Western Medicine, Nanjing University of Chinese Medicine, Nanjing, 210023 China; 4 The First School of Clinical Medicine, The Affiliated Hospital of Nanjing University of Chinese Medicine, Nanjing, 210029 China

**Keywords:** Worenine, Warburg effect, Colon cancer, HIF-1α, Glycolysis

## Abstract

**Background:**

Some natural compounds inhibit cancer cell growth in various cancer cell lines with fewer side effects than traditional chemotherapy. Here, we explore the pharmacological effects and mechanisms of worenine (isolated from *Coptis chinensis*) against colorectal cancer.

**Methods:**

The effects of worenine on colorectal cancer cell proliferation, colony formation and cell cycle distribution were measured. Glycolysis was investigated by examining glucose uptake and consumption, lactate production, and the activities and expressions of glycolysis enzymes (PFK-L, HK2 and PKM2). HIF-1α was knocked down and stimulated in vitro to investigate the underlying mechanisms.

**Results:**

Worenine somewhat altered the glucose metabolism and glycolysis (Warburg effect) of cancer cells. Its anti-cancer effects and capability to reverse the Warburg effect were similar to those of HIF-1α siRNA and weakened by deferoxamine (an HIF-1α agonist).

**Conclusion:**

It is suggested that worenine targets HIF-1α to inhibit colorectal cancer cell growth, proliferation, cell cycle progression and the Warburg effect.

**Supplementary Information:**

The online version contains supplementary material available at 10.1186/s11658-021-00263-y.

## Background

Colorectal cancer is the third most common cancer and the fourth leading cause of cancer-associated death globally [1, 2]. Conventional chemotherapy has shown no significant benefit in improving overall survival rates and it has proven to increase patients’ chronic pain. Surgery is combined with chemotherapy has been employed clinically, but the results were far from satisfactory [3]. Therefore, it is essential to develop novel anti-colorectal cancer agents with lower toxic and greater efficacy.

**Fig. 1:**
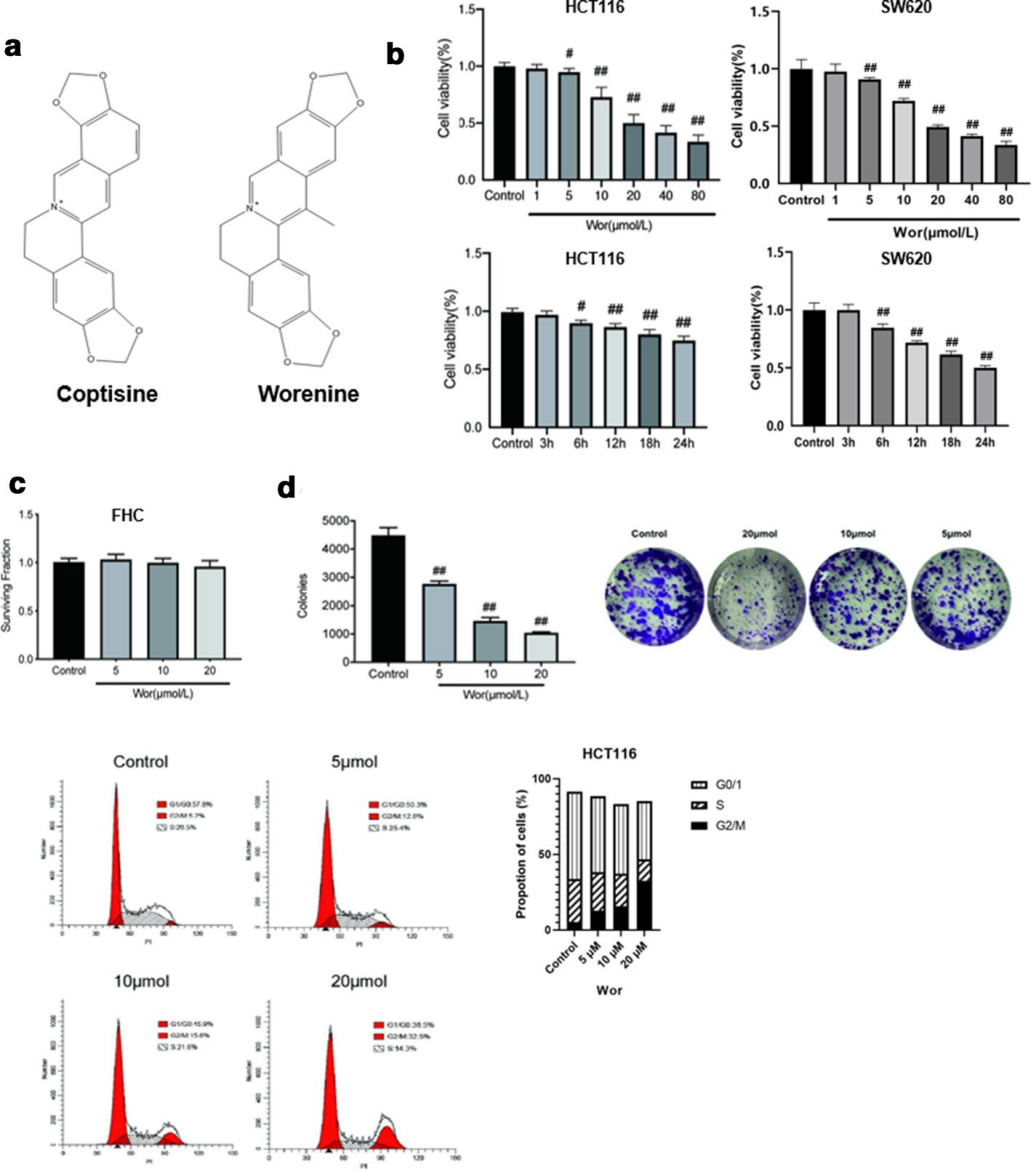
Worenine reduces cell viability and impairs cell cycle progression. **a** The chemical structures of coptisine and worenine. **b** HCT116 and SW620 cells (1 × 10^4^ cells per well) were treated with various concentrations of worenine (0, 1, 5, 10, 20, 40 or 80 μM) for 24 h. HCT116 and SW620 cells (1 × 10^4^ cells per well) were treated with 20 μM worenine for 3, 6, 12, 18 or 24 h. Cell viability was detected using the MTT assay. **c** FHC cells were treated with worenine for 24 h at the indicated concentrations. **d** The colony formation rates of HCT116 cells treated with various concentrations of worenine (5, 10 or 20 μM) for 24 h. The percentage values are based on comparison with the control group. **e** Cell cycle profiles of HCT116 cells treated with various concentrations of worenine (5, 10 or 20 μM) for 24 h. The data are means ± SEM. ^#^p < 0.05, ^##^p < 0.01 vs. control group

The dried rhizome of *Coptis chinensis* (Chinese goldthread, family Ranunculaceae) has been reported to possess anti-tumor activities in vitro and in vivo [4, 5]. It contains a range of bioactive ingredients, including ferulic acid, berberine, palmatine, magnoflorine, coptisine and worenine [6]. Coptisine showed anti-tumor activities against colon cancer through the induction of apoptosis without toxic symptoms [7]. Worenine is an isoquinoline alkaloid that has a similar chemical structure to coptisine (Fig. 1a) [8]. Therefore, we hypothesized that worenine might be a promising low toxicity compound for colorectal cancer treatment.

Unlike normal cells, most cancer cells exhibit an energy metabolism disorder that contributes to their growth and proliferation. They also display the Warburg effect: active glucose uptake and glycolysis under aerobic conditions [9]. Although the mechanisms of the Warburg effect have not been fully elucidated, multiple factors are known to regulate it, including mitochondrial oxidative phosphorylation, glycolysis enzymes, oncogenes, signal transduction pathways and the tumor microenvironment.

Hypoxia-inducible factor 1α (HIF-1α) is a key mediator of the Warburg effect in tumor cells. It activates the transcription of genes that encode proteins in oxygen-independent energy production pathways. It can promote cell glycolysis in the tumor microenvironment under hypoxic conditions by upregulating the expression of glycolytic enzymes, such as glucose transporters 3 (GLUT3), phosphofructokinase 1 (PFK1), hexokinase 2 (HK2), lactate dehydrogenase A (LDHA) and pyruvate kinase M2 (PKM2) [10].

Here, we assessed the effect of worenine on cell proliferation, cell cycle distribution and colony formation in colorectal cancer. Then we investigated glycolysis by measuring glucose uptake and consumption, lactate production, and the expressions of the glycolysis enzymes GLUT3, LDHA, PFK-L, HK2 and PKM2. We found that worenine altered the Warburg effect in the cancer cells. Using HIF-1α knockdown and stabilization, we demonstrated that the anti-colorectal cancer effects of worenine are associated with HIF-1α-modulated glycolysis. This is the first investigation of the in vitro regulatory impact of worenine on the Warburg effect in colorectal cancer.

## Materials and methods

### Cell culture

Worenine with a purity of at least 97% was obtained from Shanghai Yuanye Bio-Technology and dissolved in dimethyl sulfoxide (DMSO). The colorectal cancer cell lines HCT116 and SW620 and the normal human colon cell line FHC (CRL-1831) were purchased from the cell bank of the Chinese Academy of Sciences (CAS). The cancer cell lines were cultured in RPMI 1640 medium (Biological Industries, Israel) containing 10% (v/v) fetal bovine serum (FBS; Thermo Fisher Scientific, USA) and 100 units per ml of streptomycin and penicillin (Sigma, USA). FHC cells were cultured in DMEM/F12 (Dulbecco’s modified Eagle medium; Gibco, New Zealand) containing 10% FBS, 2.5% L-Glutamine and 100 units per ml of penicillin and streptomycin. All cells were incubated in a humidified incubator at 37 °C with 5% CO_2_.

### MTT test

The effect of worenine treatment on the cell viability of HCT-116 cells was determined using a Beyotime MTT test kit (C0009) according to the manufacturer’s instructions. Cells were seeded into 96-well plates and cultured for 24 h, followed by 24 h of worenine treatment. Then, 20 μl/well of MTT (5.0 mg/ml) was added and cultured for 4 h. The cell supernatant was removed and replenished with 200 μl DMSO to dissolve the formazan precipitate. The UV absorption at 490 nm was measured using a microplate reader (Elx800, BioTek, USA).

### Colony formation test

A colony formation test was performed to further assess the effect of worenine on cell viability. HCT-116 cells were seeded into 6-well dishes and incubated for 24 h. Afterwards, cells were treated with 5, 10 and 20 μM worenine for 24 h. The cell culture was substituted with fresh medium every 2 days. After 2 weeks, colonies were fixed in methanol and stained with 0.1% crystal violet. Colonies with at least 50 cells were counted under an inverted microscope.

### Cell cycle analysis

Propidium iodide (PI) RNase staining solution (4087S, CST) was used for the cell cycle assay. HCT-116 cells were incubated with worenine for 24 h. Afterwards, the cells were washed once with phosphate-buffered saline (PBS) and fixed overnight with 500 μl chilled 70% ethanol at 4 °C, then washed again with PBS. They were then suspended with the staining solution as described previously and incubated in darkness at 4 °C for 30 min. Finally, the cells were quantified using FACS-Calibur flow cytometry (BD Biosciences, USA).

### Assay of the glucose uptake and consumption levels and lactate production level

After worenine treatment for 72 h, the medium was collected for lactate and glucose concentration analysis. The lactate content in the media were tested according to the manufacturer’s instructions for the Lactic Acid Production Detection kit (Nanjing Jiancheng Bioengineering Institute, China). To evaluate the glucose uptake and consumption, a Glucose Uptake Colorimetric Assay Kit (Sigma, USA) and a Glucose Assay Kit (Solarbio Science & Technology, China) were respectively used according to the manufacturers’ instructions.

### Western blot analysis

Cells were rinsed three times with ice-cold PBS, lysed in RIPA buffer for 30 min at 4 °C, and then centrifugated at 8000 g for 10 min at 4 °C. The protein concentration was measured using the Bradford protein assay. The membrane and nuclear proteins of the HCT116 cell were extracted and isolated using a Cell membrane–Cytoplasm–Nucleoprotein Stepwise Extraction Kit (BB31042, Bestbio). Using sodium dodecylsulphate-polyacrylamide gel electrophoresis (SDS-PAGE), 50 μg of protein was purified and then transferred to polyvinylidene difluoride (PVDF) membrane. Following blocking with 5% non-fat milk (0.05% Tween 20 Tris-saline) at room temperature for 2 h, the membranes were incubated with primary antibodies (Abcam, USA) against HIF-1α (1:1000), p-VHL (1:1000), β-actin (1:5000), lamin B1 (1:1000), Na/K-ATPase (1:1000), PFK-L (1:10,000), HK-2 (1:1000), PKM-2 (1:1000), GLUT3 (1:1000) and LDHA (1:1000) at 4 °C overnight. The membranes were then incubated with secondary antibodies (1:10,000; Proteintech Group, USA), then washed three times with TBST. Bands were visualized with an enhanced chemiluminescence system (Pierce Biotechnology) and measured using ImageJ software (National Institutes of Health). Each of the experiments was independently repeated three times.

### RT-PCR analysis

The expression levels of PFK-L and HK-2 mRNA in cells were detected using RT-PCR using our laboratory protocol [11]. Briefly, total RNA was extracted using Trizol (Invitrogen, USA), according to the manufacturer’s instructions. RT-PCR was performed using the StepOne Real- Time PCR system (Applied Biosystems, USA), and the 2^−∆∆Ct^ method was utilized to calculate the relative expressions of the targets. β-actin served as the internal control.

The primer sequences were designed by Sangon Biotech (China) and were: HK-2 forward, 5′-CGACAGCATCATTGTTAAGGAG-3′ and reverse, 5′-GCAGGAAAGACACATCACATTT-3′; and PKM2 forward, 5′-CCTCCTTCAAGTGCTGCAGT-3′ and reverse, 5′-TCATGGCAAAGTTCACCCGG-3′. The experiments were repeated in triplicate.

### Immunofluorescence assay

After treatment with worenine, cells were fixed in 4% paraformaldehyde for 15 min, washed 3 times in PBS and blocked with 5% BSA for the indicated times. Next, the prepared cells were dyed at 4 °C with anti-GAPDH (1:50), anti-PFKL (1:100) and anti-HK-2 (1:100) overnight. After being rinsed 3 times in PBS, the cells were incubated with FITC‐labeled secondary antibodies (1:100) for 1 h at 37 °C in the dark. To label the nuclei, cells were counter-stained with DAPI (Beyotime Institute of Biotechnology, China). Images were acquired using a fluorescent microscope (Leika, Germany). The results were obtained from triplicate experiments.

### ELISA assay

The concentrations of protein were evaluated using the Human Hexokinase 2 (HK-2) ELISA Kit, Human Phosphofructokinase L (PFK-L) ELISA Kit, and Human Pyruvate Kinase Isozymes M2 (PKM2) ELISA Kit (Bio-Swamp, China). The samples for ELISA were prepared as follows: the treated HCT-116 cells were collected and centrifuged at 8000 g for 15 min at 4 °C and the cell supernatant was stored at − 20 °C. The absorbance of all the samples was measured using an ELISA reader (Bio-Tek, USA), and the data were processed using Bio-Tek software.

### RNAi treatments

siRNA targeting HIF-1α was purchased from Burson-Marstor Bioengineering (China). The antisense sequence of the siRNA against HIF-1α is 5'-AGTTCACCTGAGCCTAATA-3'. The HIF-1α siRNA (100 nM) was transfected into HCT116 cells using Lipofectamine 2000 according to the manufacturer's instructions (Invitrogen Life Technologies, USA).

### Enzyme activity analysis

The hexokinase (HK) activity was quantified using a hexokinase activity assay kit (BC0740; Solarbio). The phosphofructokinase (PFK) and pyruvate kinase (PKM) activities were respectively measured with a phosphofructokinase activity detection kit (BC0535; Solarbio) and pyruvate kinase assay kit (ab83432, Abcam). Generally, cells (5 × 10^6^) in 1 ml of extracting solution were broken using ultrasonication on ice and then centrifuged at 8000 g, 4 °C, for 10 min. The supernatant was measured spectrophotometrically at 340 nm. The HK, PFK and PKM2 activities were calculated based on the protein concentration in the sample using a BCA assay (Beyotime, China).

### Statistical analysis of data

Statistical analysis was done with SPSS 20.0 software. All data are expressed as the means ± SEM and were obtained from multiple independent experiments. One-way ANOVA was used to detect differences among different groups and p < 0.05 was considered significant.

## Results

### Worenine inhibits colorectal cancer cell viability and causes cell cycle arrest

The MTT assay was used to examine the effect of worenine on two human colorectal cancer cell lines, HCT116 and SW620. Worenine was found to repress colorectal cancer cell viability at concentrations ranging from 5 to 20 μM (Fig. 1b). The IC_50_ for HCT116 and SW620 cells were 18.30 and 15.19 μM, respectively. Worenine also affected the cell viability of colorectal cancer cells in a time-dependent manner. Importantly, it was shown to have anticancer effects without causing significant cell death of normal FHC human colon cells (Fig. 1c), indicating that worenine is likely to be a safe anti-colorectal cancer compound.

A cell colony formation experiment was used to further explore the long-term anti-colorectal cancer effects of worenine. The number of cell colonies decreased with increasing worenine doses (Fig. 1d, Additional file 1: Figure S1A).

In addition, worenine triggered cell cycle arrest and increased the G2-to-M cell ratio (enhanced the G2/M peak; Fig. 1e, Additional file 1: Figure S1B). These results reveal that worenine can inhibit cell viability and induce cell cycle arrest in colorectal cancer cells in vitro.

### Worenine significantly dampens glycolysis in HCT116 cells

**Fig. 2:**
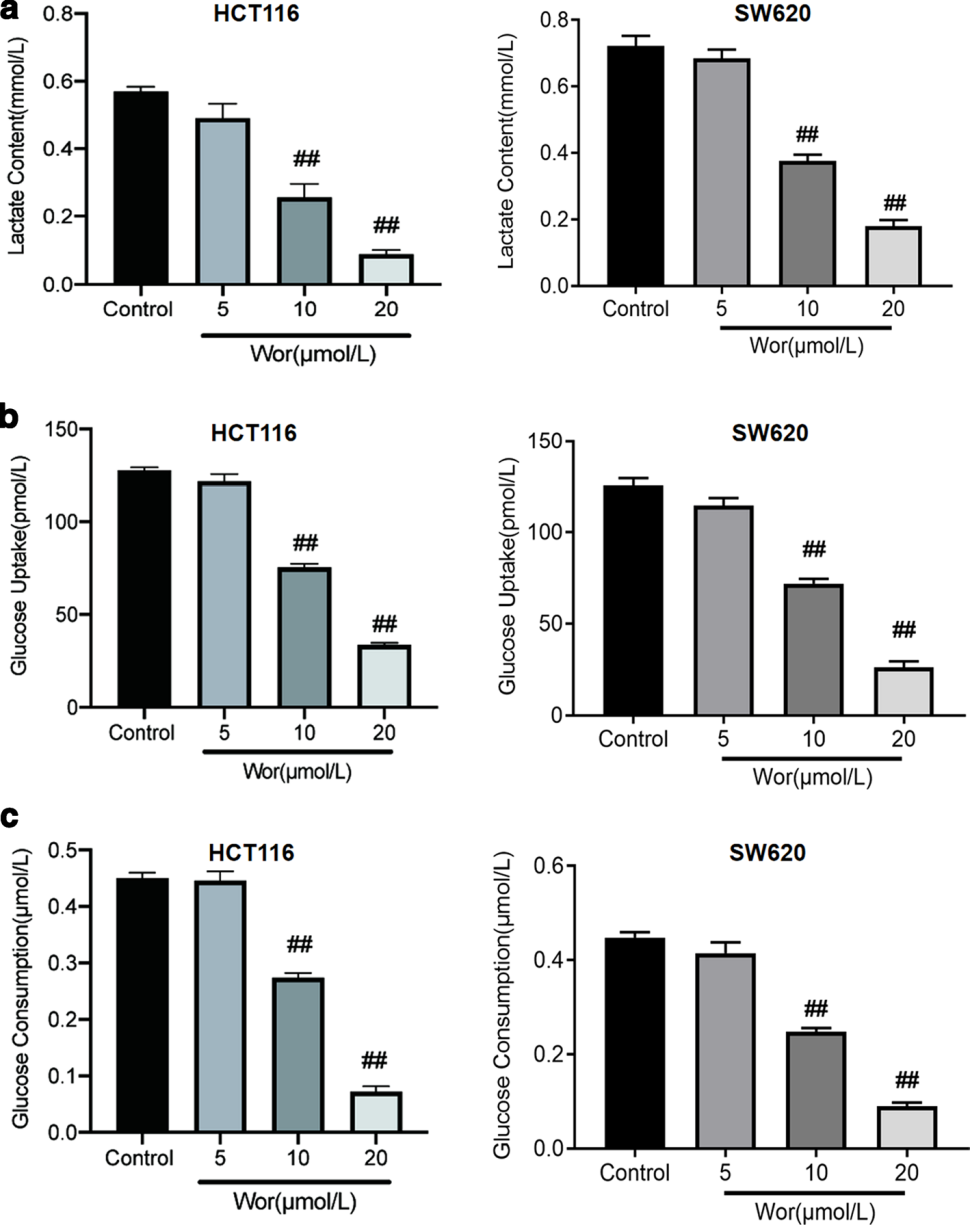
Glucose consumption, glucose uptake and lactate release for colorectal cancer cells after worenine treatment. **a** The lactate level in the supernatant was determined using a lactic acid production detection kit. **b** Glucose uptake was evaluated using a glucose uptake colorimetric assay kit. **c** Glucose consumption was evaluated using a glucose assay kit. The data are means ± SEM. ^#^p < 0.05, ^##^p < 0.01 vs. control group

To explore the effect of worenine on colorectal cancer cell glycolysis, we measured worenine-induced changes in the levels of lactic acid production as well as glucose uptake and consumption. In both HCT116 and SW620 cells, worenine treatment significantly suppressed lactic acid production and glucose uptake and consumption at concentrations of 10 μM and 20 μM. (Fig. 2a–c). These results indicated that the Warburg effect of colorectal cancer cells is weakened by worenine. However, worenine had no significant effect on the glycolysis of FHC cells compared with the control cells (Additional file 2: Figure S2).

### Worenine inhibits key enzymes in glycolysis

**Fig. 3:**
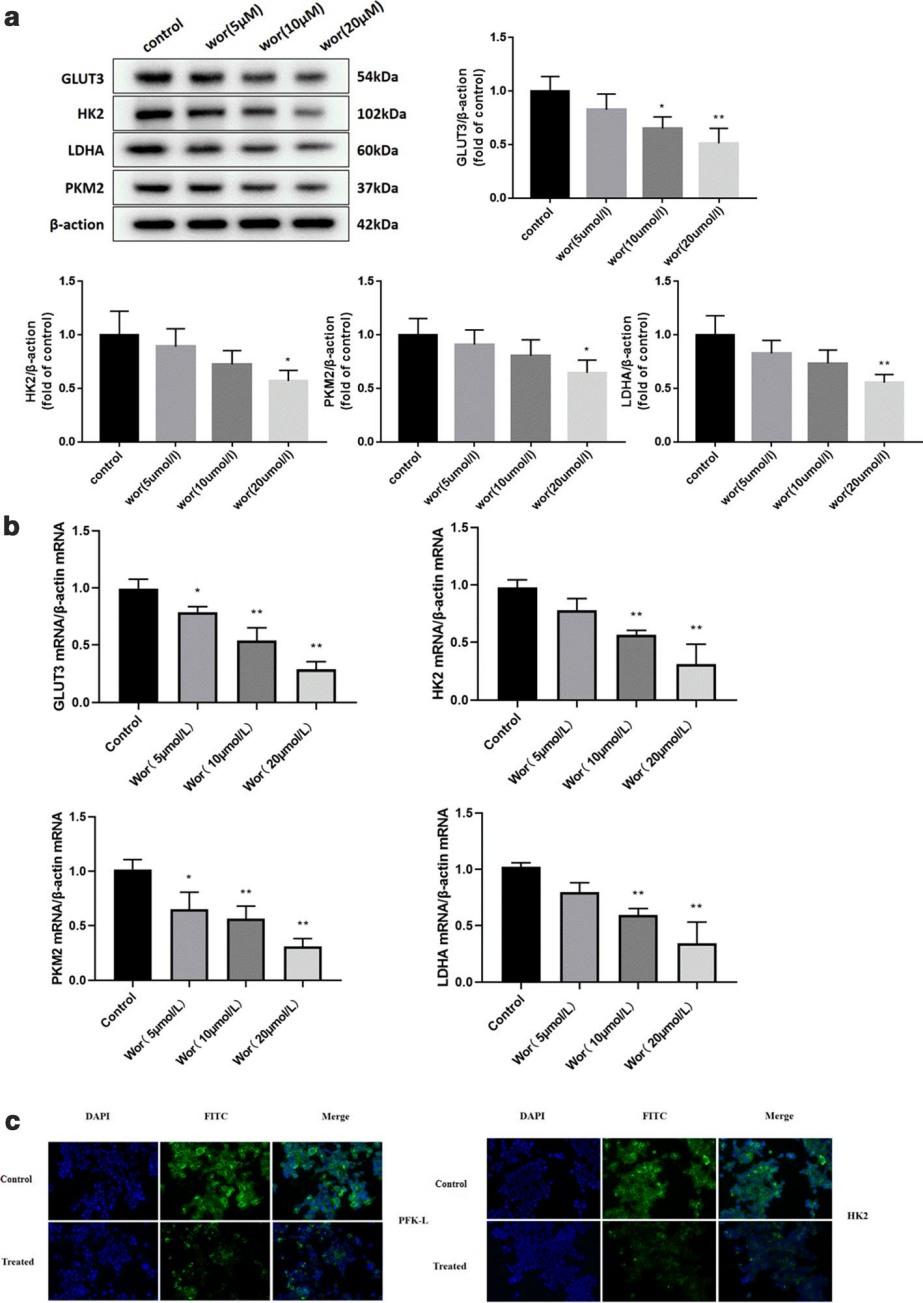
Key glycolytic enzymes are inhibited by worenine. HCT116 cells were treated with 5, 10 or 20 μM worenine for 24 h. **a** The protein expressions of GLUT3, HK2, PKM2 and LDHA were determined using western blotting. **b** he results for the quantitative RT-PCR analysis of the mRNA expressions of GLUT3, HK2, PKM2 and LDHA in worenine-treated cells. **c** HK-2 and PFK-1 expression were evaluated using immunofluorescence. Cells were co-stained with DAPI to visualize the nuclei. The data are means ± SEM. ^#^p < 0.05, ^##^p < 0.01 vs. control group

We further investigated the mechanism by which worenine inhibits glycolysis by examining several key glycolytic enzymes that are overexpressed in colorectal cancer cells: GLUT3, HK-2, PFK-L, PKM2 and LDHA. High doses of worenine significantly decreased the protein and mRNA levels of GLUT3, HK-2, PFK-L, PKM2 and LDHA (Fig. 3a–c, Additional file 3: Figure S3A andB). The enzyme activities of PFK, HK, PKM were also measured (Additional file 3: Figure S3C). The results indicate that the activity of PKM in HCT116 cells decreased with increasing worenine concentration. The activity of HK went up slightly when treated with worenine. This may be due to the slight reduction in the inhibition effect of its product, G-6-P. The activity of PFK remained unchanged. These results suggest that worenine affects glycolysis via the regulation of key glycolytic enzymes.

### Worenine promotes HIF-1α degradation

**Fig. 4:**
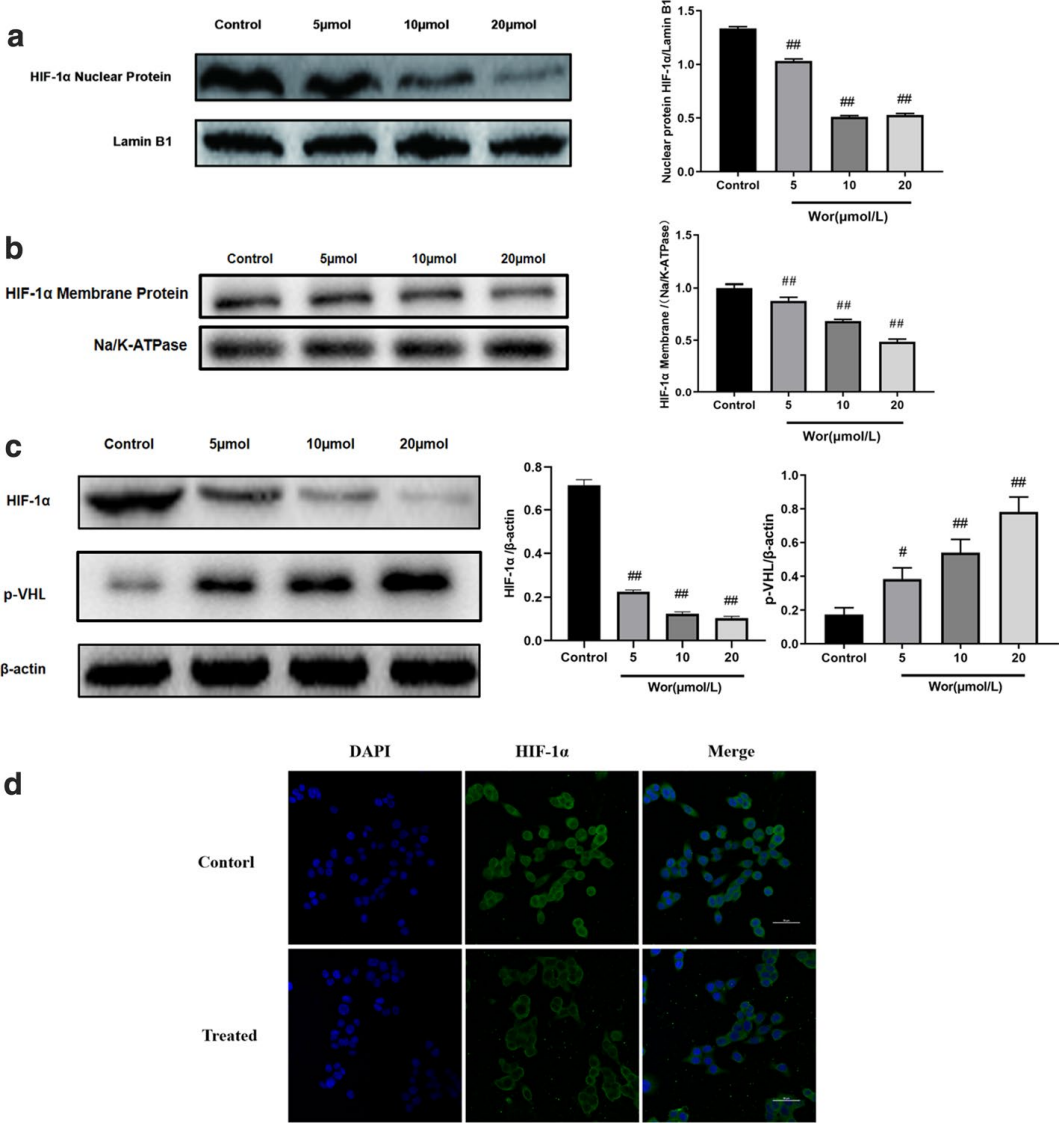
Worenine promotes the degradation of HIF-1α and inhibites HIF-1α entry into the nucleus. HCT116 cells were treated with various concentrations of worenine (5, 10 and 20 μM) for 24 h. **a** The effect of worenine on nuclear HIF-1α expression in cells was investigated via western blotting analyses of nuclear protein extracts. **b** The effect of worenine on membrane HIF-1α expression in cells was investigated via western blotting analyses of membrane protein extracts. **c** After treatment with a protein synthesis inhibitor, the protein expression levels were determined using western blotting. **d** Immunofluorescence staining of HIF-1α with or without worenine. The nucleus of the HCT116 cells was stained by DAPI. The data are means ± SEM. ^#^p < 0.05, ^##^p < 0.01 vs. control group

It is widely known that HIF-1α is overexpressed in colorectal cancer cells and could increase the activity of glycolytic genes, such as GLUT, HK2 and PKM-2, thus evoking glycolysis. Western blot results of nuclear and membrane fractions of HCT116 cells revealed that worenine inhibited nuclear HIF-1α more than the membrane HIF-1α (Fig. 4a and b). Further results (Fig. 4c) suggest that Worenine elevated pVHL levels and decreased HIF-1α protein expression after treatment with the protein synthesis inhibitor. The fluorescent images showed similar results (Fig. 4d). The fluorescent puncta of HIF-1α was weakened by worenine. Based on these results, we conjecture that Worenine could decrease the HIF-1α level in HCT116 cells by preventing HIF-1α nuclear translocation and promoting HIF-1α degradation.

### Worenine exerts an inhibitory effect on glycolysis and cell viability through HIF-1α signaling

**Fig. 5:**
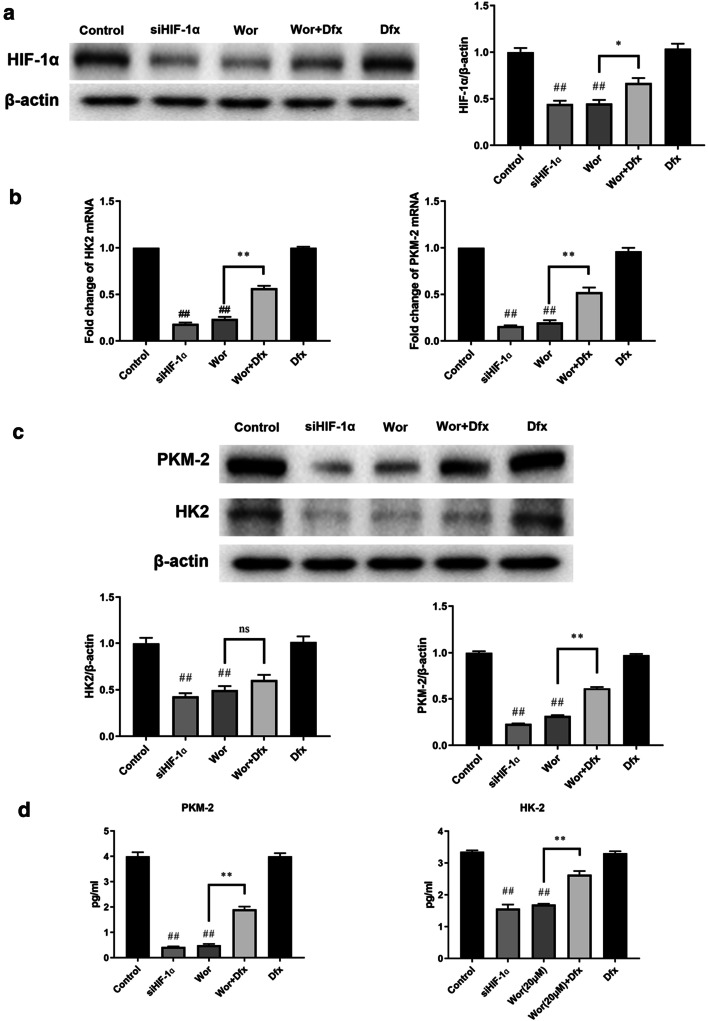
Worenine inhibits HIF-1α and further blocks HK-2 and PKM2 expression. HCT116 cells were treated with worenine (Wor; 20 μM), desferrioxamine (Dfx; 30 μM), siRNA of HIF-1α (siHIF-1α), and worenine combined with desferrioxamine (Wor + Dfx) for 24 h. DMSO-treated cells were used as a control. **a** The protein expression of HIF-1α was assayed via western blotting. **b** The mRNA expressions of PKM-2 and HK2 were determined using real-time PCR. **c** GLUT3, HK2, PKM2 and LDHA protein expressions were determined using western blotting. **d** The concentrations of HK-2 and PKM2 were determined using ELISA. The data are the means ± SEM. ^##^p < 0.01 vs. control group. *p < 0.05 and **p < 0.01 vs. worenine group

To determine the role of HIF-1α in worenine-induced effects on the glycolysis and cell growth of HCT116 cells, HIF-1α siRNA (siHIF-1α) and desferrioxamine (Dfx) were respectively used to inhibit and stabilize HIF-1α. The expression of HIF-1α protein was knocked down by siHIF-1α (Fig. 5). Similarly to worenine treatment, siHIF-1α downregulated the mRNA and protein levels of HK-2 and PKM2. Desferrioxamine (Dfx) is an iron chelator that can stabilize HIF-1α without altering HIF-1α mRNA expression. With the stabilization of HIF-1α by Dfx, the decrease in HK-2 and PKM2 expression induced by worenine treatment was reversed. In addition, Dfx (30 μM) alone did not affect HIF-1α, HK-2 and PKM2 expression.

The effects of worenine on glucose uptake, glucose consumption and lactate generation were mimicked by siHIF-1α and weakened by Dfx treatment (Additional file 4: Figure S4A–C). Furthermore, a remarkable decrease in cell viability was observed after treatment with 20 μM worenine or siHIF-1α, compared with control group (Additional file 4: Figure S4D). Nevertheless, Dfx treatment hindered the worenine-induced decrease in cell viability, but Dfx (30 μM) alone did not affect cell viability and glycolysis. We concluded that worenine inhibits the Warburg effect and cell growth of HCT116 cells by decreasing the levels of HIF-1α.

## Discussion

Worenine isolated from *Coptis chinensis* was discovered to exhibit anti-cancer activity in HCT116 and SW620 cells in our preliminary MTT-based evaluations. However, the effects and mechanisms of worenine against cancer have not been fully elucidated. Here, we found that worenine inhibited colorectal cancer cell growth, proliferation, cell cycle progression and the Warburg effect by targeting HIF-1α in vitro.

The Warburg effect refers to cancer cells prioritizing glycolysis rather than oxidative phosphorylation as the means of metabolizing glucose, even in the presence of oxygen, producing lactate as the final product. This metabolic transition helps increase tumor cell proliferation because aerobic glycolysis produces enough energy from limited resources for cancer cells to survive and enable metabolic intermediates to shift from energy production to bio-synthesis [12]. This occurs because the expressions of different glycolytic enzyme isoforms can be changed in cancer cells by certain transcriptional factors, such as HIF-1α.

In colorectal cancer, tumorigenic cells upregulate GLUT3, HK2, PFK-L, PKM2 and LDHA expression, enhancing glycolytic flux and increasing glucose uptake [13]. GLUT1 is the most widely overexpressed transporter in cancer cells and it has been used as an indicator for poor colorectal cancer prognosis [14]. The cancer-specific isoform PKM2 confers tumorigenic cells with the ability to withstand oxidative stress. PFK-1 is a 380 KDa homo- or hetero-tetramer found in all tissues. It consists of three isoforms: PFK-L, PFK-P and PFK-M. However, HIF-1α only increases the expression of the PFK-L isoform [15]. HK2 is one of the rate-limiting enzymes involved in the step of producing glucose-6-phosphate [16]. It plays an essential role in the regulation of glycolytic flux. A high level of LDHA correlates with aggressive forms of several different tumor types, including colorectal cancer [17]. These molecules, which are involved in glycolysis, can be ideal therapeutic targets for hypoxic and glycolytic tumors [15].

Interestingly, worenine induced downregulations of PFK-L, HK2 and PKM2, indicating that the level of glycolysis, cellular energy production and macromolecular biosynthesis were declining in the tumor cells. In other words, the Warburg effect was reversed by worenine to a certain extent.

In addition, the high level of glycolytic enzymes widely observed in cancer cells has been proven to be essential for tumor cells to grow [18]. These levels are closely associated with poor overall survival in cancer patients. We found that glucose uptake and consumption as well as lactate production in HCT116 and SW620 cells were reduced in a dose-dependent manner after worenine treatment.

Hypoxia is a feature of solid tumors, including colorectal cancer. It regulates the cancer cell metabolism reprogramming that promotes tumor cell growth, invasion and infiltration. HIF-1 is a major transcription factor under hypoxia. It mediates glycolysis by regulating the expression of glycolytic genes [18]. It is a basic helix-loop-helix heterodimeric transcription factor composed of alpha (α) and beta (β) subunits [19, 20]. HIF-1α expression persists in cells under normoxia, but it is unstable as it is degraded through the ubiquitin–proteasome (26S) pathway. The ubiquitin E3 ligase complex mediates this degradation, where the von Hippel–Lindau tumor suppressor protein (pVHL) can associate with oxygen-dependent destruction domain(s) on the subunit. However, hypoxia prevents hydroxylation, thus preventing the tumor-suppresive protein from binding to proline on HIF-1α. Consequently, pVHL is inactivated, which prevents HIF-1α from becoming a target for 26S proteasome degradation [21]. This allows HIF-1α to bind to its corresponding HIF-1β subunit to form the heterodimer HIF-1 transcription factor complex. Then, this complex enters the nucleus and binds to the hypoxia response elements (HREs) on specific strands of DNA, in order to target genes that code for supporting tumor cell survival and increasing proliferative capacity and metastasis potential [22, 23].

Here, worenine decreased the HIF-1α level by activating p-VHL. Recent studies have suggested that HIF-1α is a reasonable therapeutic target for cancer treatment [24]. HIF-1α inhibition by RNA interference or inhibitors could reduce tumor cells growth and metastasis [25, 26]. We found that worenine improved the glycolysis in colorectal cancer cells in a manner similar to HIF-1α siRNA.

Based on this evidence, we hypothesize that worenine exerts anti-colorectal cancer activity by targeting HIF-1α. To verify our hypothesis, desferrioxamine (Dfx) was used to stimulate HIF-1α protein aggregation. It is an iron chelator that increases intracellular HIF-1α levels as the upregulation of HIF prolyl hydroxylases (HIF-PH) requires iron [27]. Dfx treatment reversed worenine-induced effects on the Warburg effect, confirming worenine-induced colorectal cancer cell death to have occurred via HIF-1α inhibition (Additional file 5).

However, since four aspects are involved in the inhibition of HIF-1α activity, including protein synthesis, HIF-1α degradation, transcriptional activity and mRNA levels, further investigation is required to reveal more detailed mechanisms underlying the anti-cancer properties of worenine [28, 29]. In addition, to sufficiently evaluate whether worenine can be a candidate for the clinical treatment of colorectal cancer, its protective effect should be further confirmed in vivo.

## Conclusion

This study showed that worenine derived from *Coptis chinensis* exerts anti-colorectal cancer function by affecting the HIF-1α-mediated glycolysis in the cells. Worenine-induced downregulation of HIF-1α was involved in this process. Our research provides new insights into the development of natural compounds as anti-colorectal cancer drugs.

## Supplementary Information

**Additional file 1: Fig. S1**. Worenine inhibits cell proliferation and impairscell cycle progression of SW620. SW620 cells were treated with worenine (0~20 μM) as indicated for 24h. A – Colony formation assay results for SW620. B – Cell cycle profile of SW620tested using flow cytometry. (n = 3)

**Additional file 2: Fig. S2.** The effect of worenine on FHC glycolysis. FHC cells were treatedwith worenine (0~20 μM) as indicated for 24h. A – The lactate level in the supernatants was determined usinga lactic acid production detection kit. B – Glucose uptake wasevaluated using a glucose uptake colorimetric assay kit. C – Glucose consumptionwas evaluated using a glucose assay kit. The data are means ± SEM. ^#^p < 0.05, ^##^p< 0.01 vs. control group.

**Additional file 3:** **Fig. S3** The effect of Worenine on glycolysis enzymes.HCT116 cells were treatedwith worenine (0~20 μM) as indicated for 24h. A – The effect of worenine treatment at different concentrationson the protein expression of PFK-L in cell lysates was determined using a westernblotting assay. B – The effect of worenine treatmentat different concentrations on the protein expression of PFK-L and HK2 in cell supernatants was determined using ELISA. C – The effect of worenine treatment at different concentrationson the activity of PFK, HK and PKM was tested with a phosphofructokinase assaykit, hexokinase assay kit and pyruvate kinase assay kit, respectively. The data are means ± SEM. ^#^p< 0.05, ^##^p < 0.01, *p < 0.05, **p < 0.01 vs. control group.

**Additional file 4:** **Fig. S4** Worenine inhibits glycolysisand cell viability by suppressing HIF-1α. HCT116 cells were treated with worenine (20 μM), desferrioxamine (30μM), siRNA of HIF-1α, and worenine combinedwith desferrioxamine. DMSO-treated cells were used as a control. The lactate level(A), the glucose uptake level (B) and the glucose consumption level (C) were measuredwith the appropriate kits. D – The cell viability of HCT116 cells was determinedusing an MTT assay. The data are means ± SEM. ^##^p < 0.01 vs. controlgroup. **p < 0.01 vs. worenine group.

**Additional file 5:** Original data.

## Data Availability

All data from this study are available in this published article.

## Abbreviations

HIF-1: Hypoxia-inducible factor-1
PFK: Phosphofructokinase
HK: Hexokinase
PKM2: Pyruvate kinase M2
Dfx: Desferrioxamine
pVHL: Von Hippel–Lindau tumour suppressor protein
