# Supplementary figures and images for: Worenine reverses the Warburg effect and inhibits colon cancer cell growth by negatively regulating HIF-1α
Source: Cell Mol Biol Lett. 2021 May 18;26:19. doi: 10.1186/s11658-021-00263-y (PMC8130299; doi:10.1186/s11658-021-00263-y)

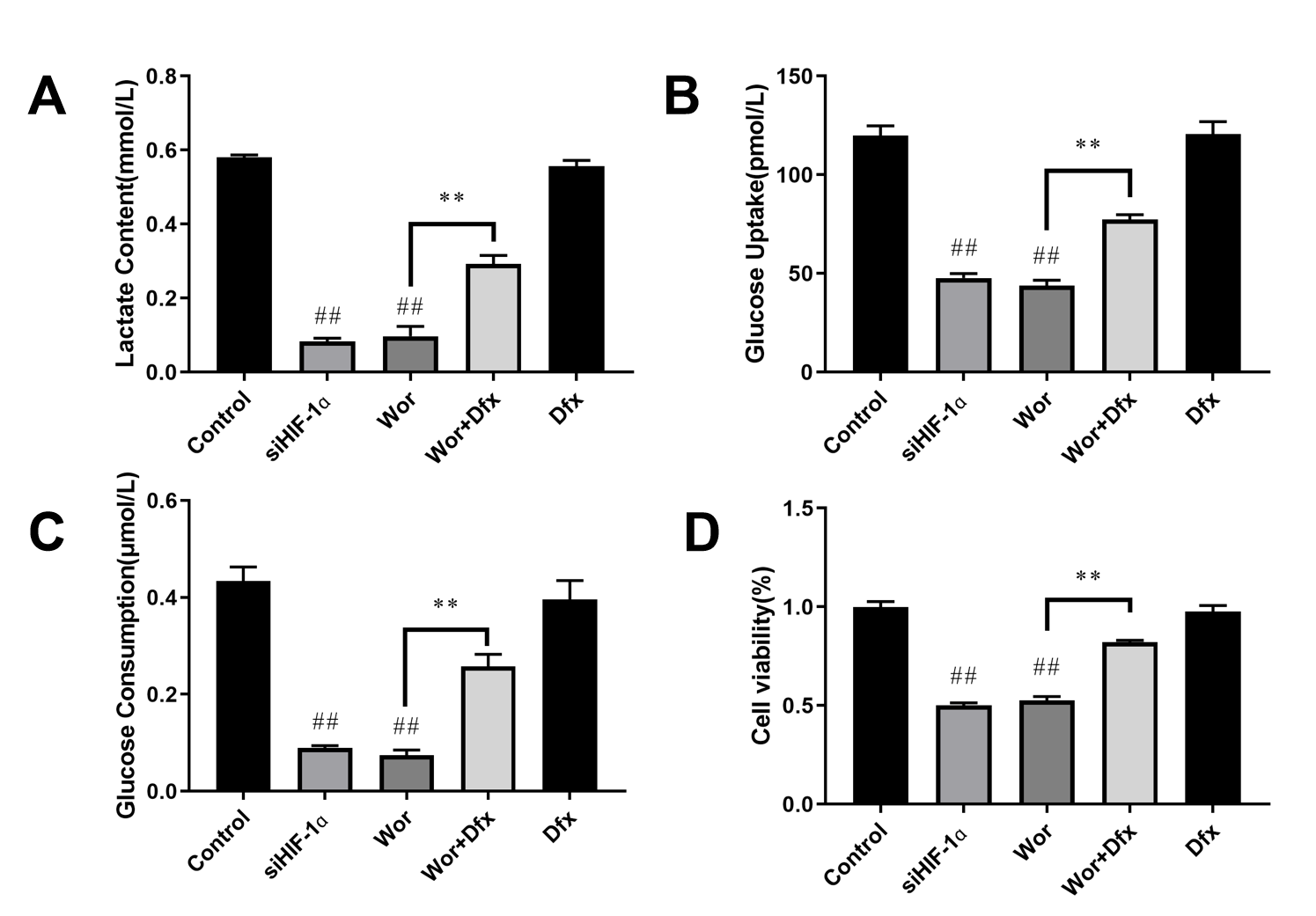

Supplement: Supplementary file 2 — Additional file 2: Fig. S2. The effect of worenine on FHC glycolysis. FHC cells were treatedwith worenine (0~20 μM) as indicated for 24h. A – The lactate level in the supernatants was determined usinga lactic acid production detection kit. B – Glucose uptake wasevaluated using a glucose uptake colorimetric assay kit. C – Glucose consumptionwas evaluated using a glucose assay kit. The data are means ± SEM. #p < 0.05, ##p< 0.01 vs. control group. [file 11658_2021_263_MOESM2_ESM.tif]

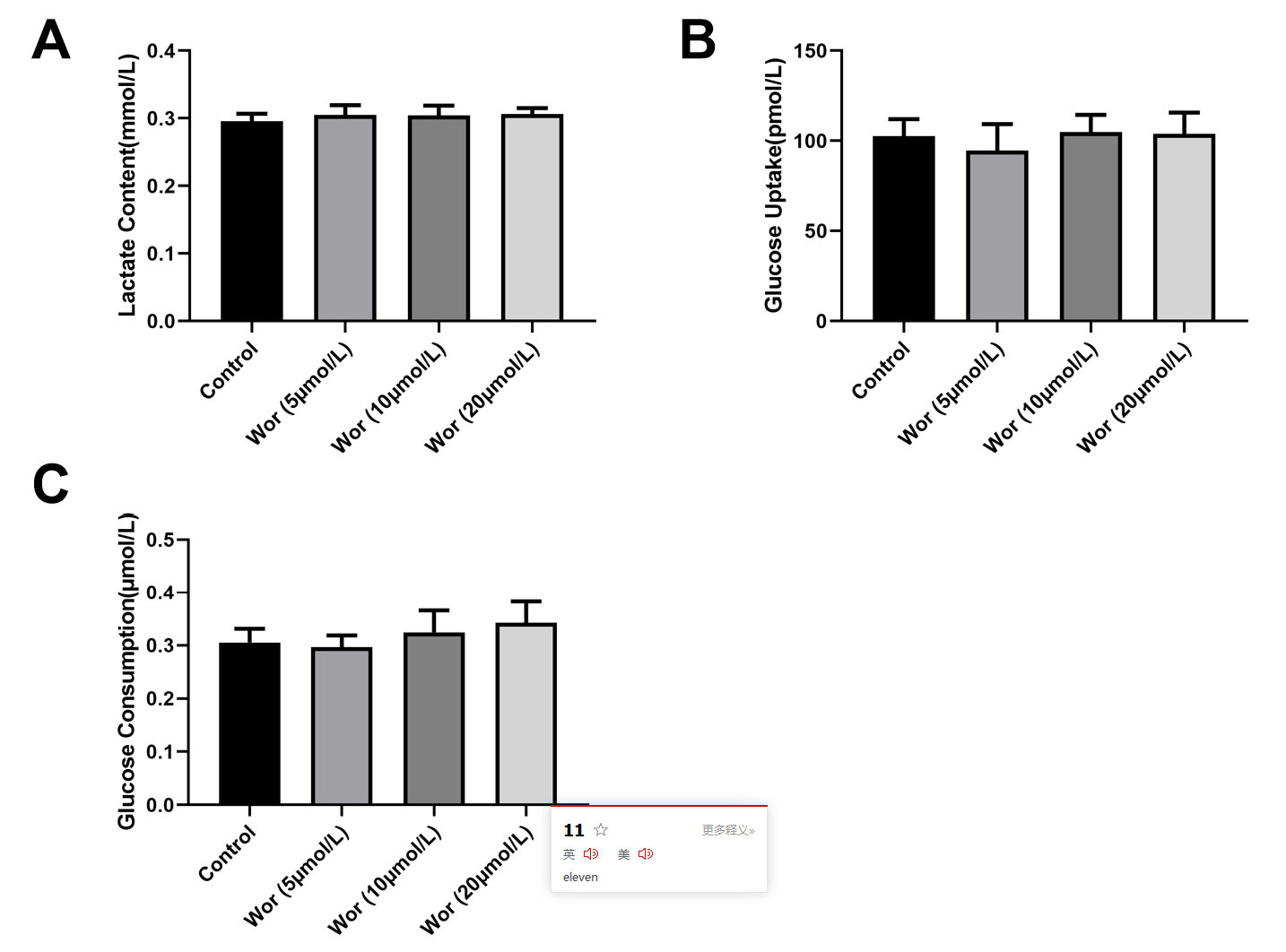

Supplement: Supplementary file 3 — Additional file 3: Fig. S3 The effect of Worenine on glycolysis enzymes.HCT116 cells were treatedwith worenine (0~20 μM) as indicated for 24h. A – The effect of worenine treatment at different concentrationson the protein expression of PFK-L in cell lysates was determined using a westernblotting assay. B – The effect of worenine treatmentat different concentrations on the protein expression of PFK-L and HK2 in cell supernatants was determined using ELISA. C – The effect of worenine treatment at different concentrationson the activity of PFK, HK and PKM was tested with a phosphofructokinase assaykit, hexokinase assay kit and pyruvate kinase assay kit, respectively. The data are means ± SEM. #p< 0.05, ##p < 0.01, *p < 0.05, **p < 0.01 vs. control group. [file 11658_2021_263_MOESM3_ESM.tif]
