# Supplementary material for: Worenine reverses the Warburg effect and inhibits colon cancer cell growth by negatively regulating HIF-1α
Source: Cell Mol Biol Lett. 2021 May 18;26:19. doi: 10.1186/s11658-021-00263-y (PMC8130299; doi:10.1186/s11658-021-00263-y)
Supplement: Supplementary file 5 — Additional file 5: Original data. [file 11658_2021_263_MOESM5_ESM.pdf]

Original Data of Western Blot

Figure 3A

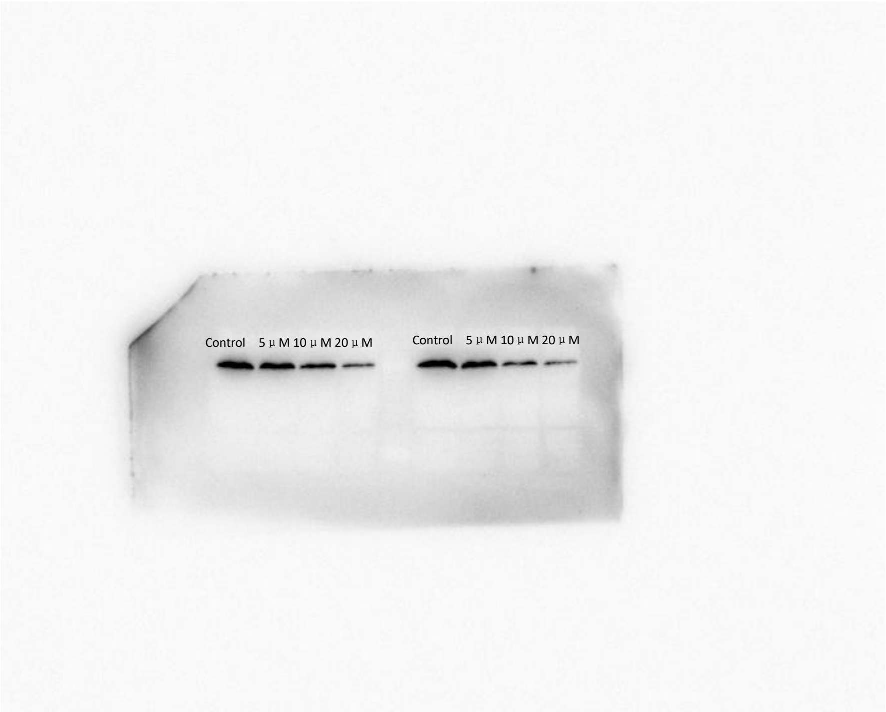

PFK-L

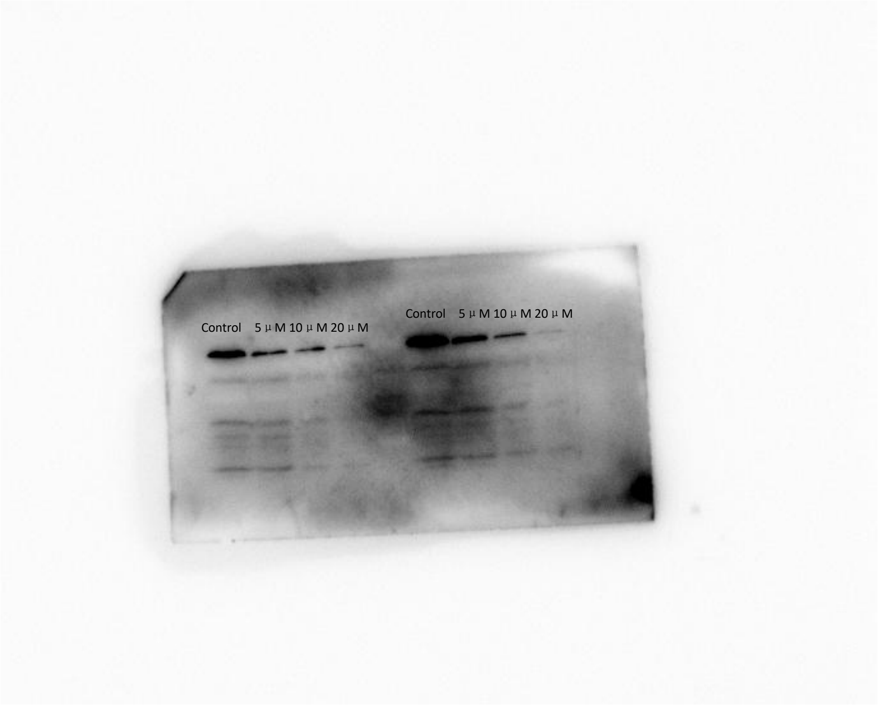

HK-2

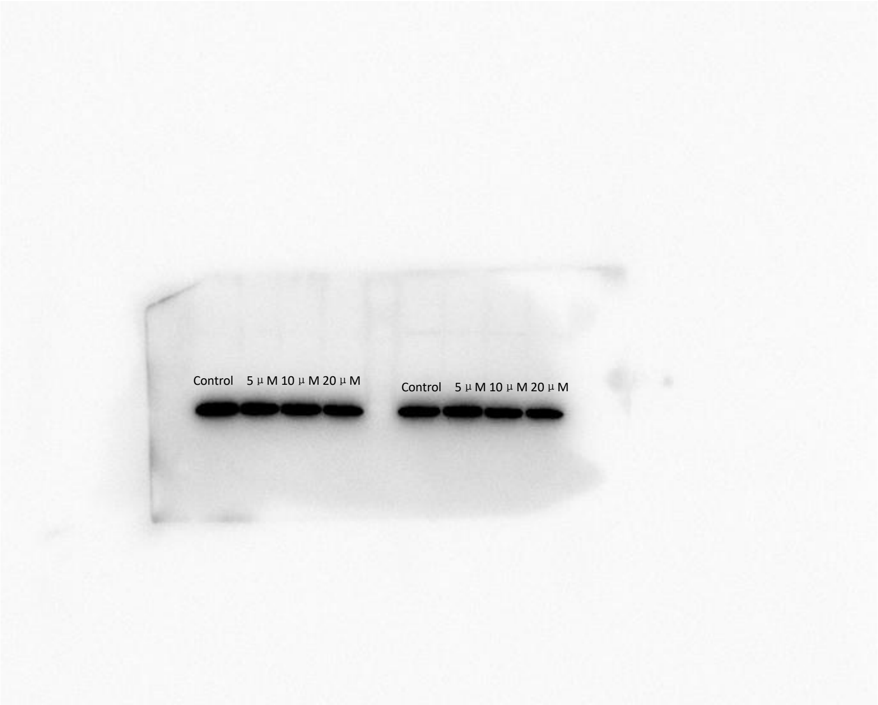

β -actin

Figure 4A

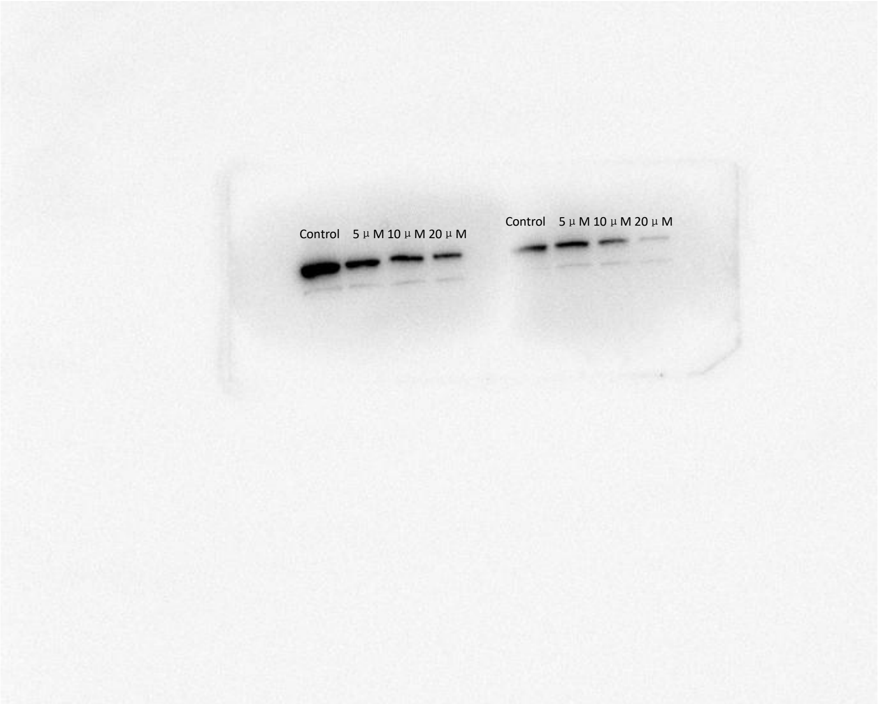

Nuclear protein HIF-1 α

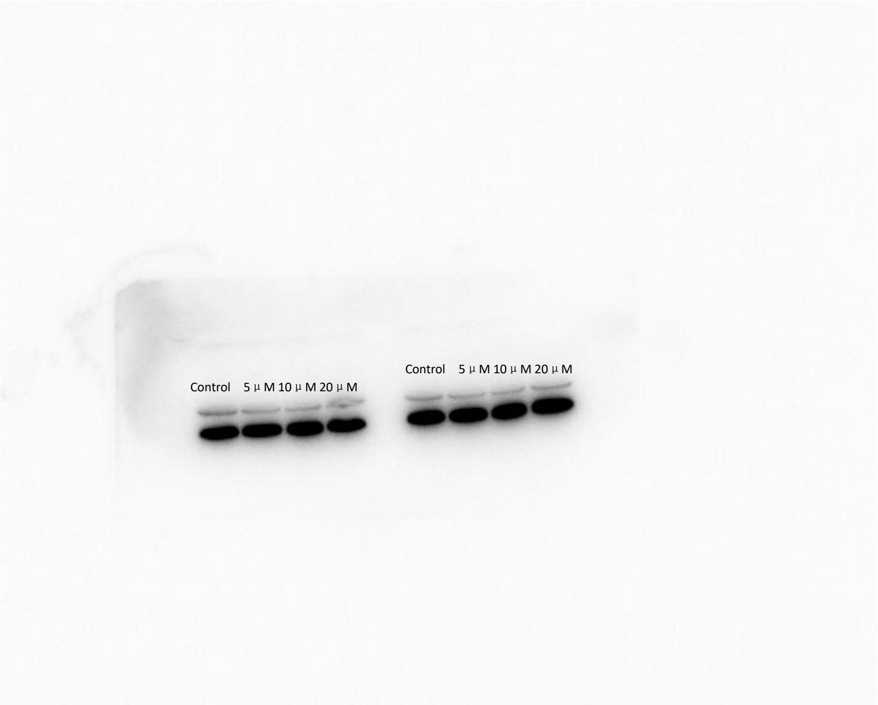

Lamin B1

Figure 4B

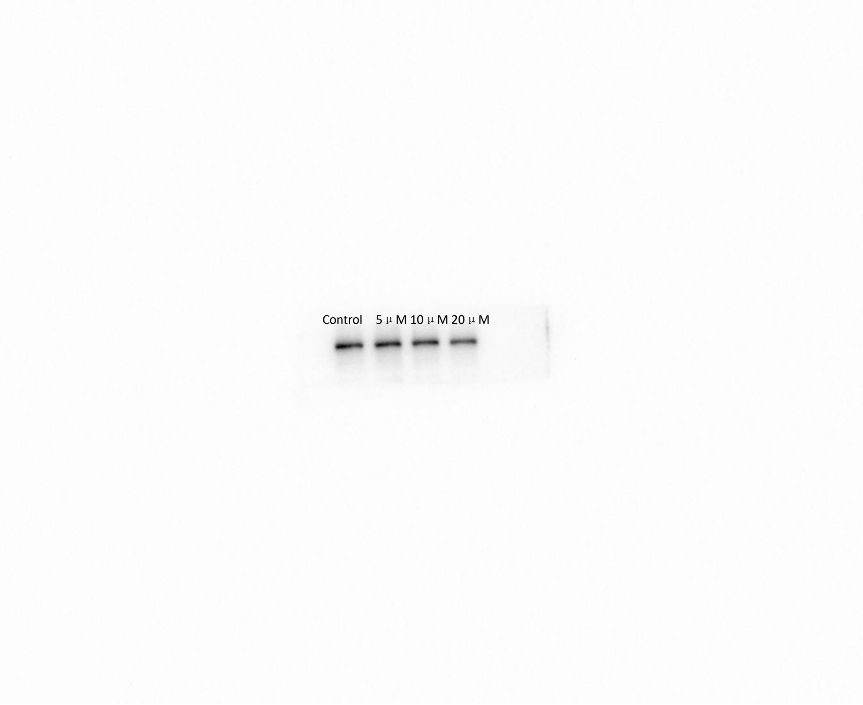

HIF-1a

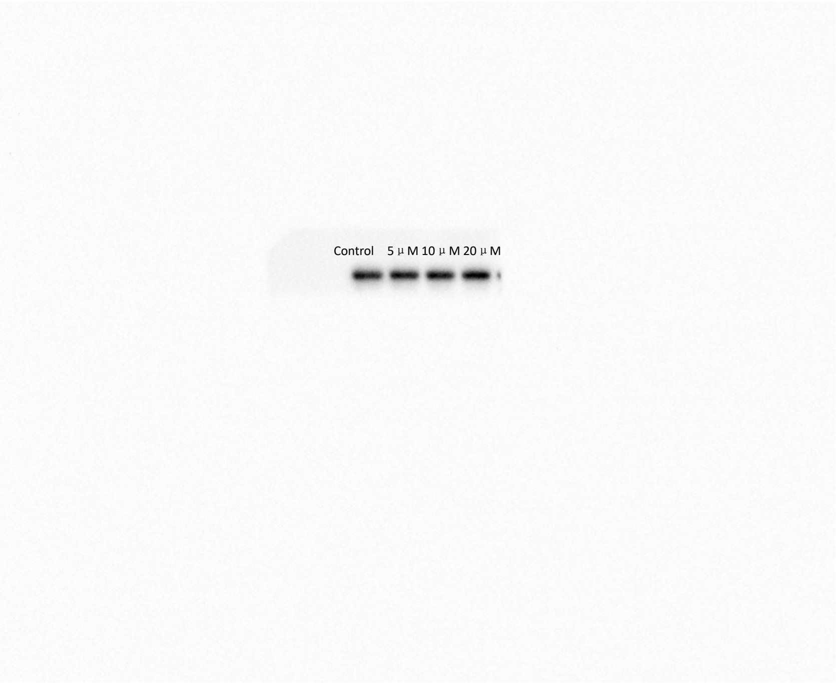

Na/K-ATPase

Figure 4C

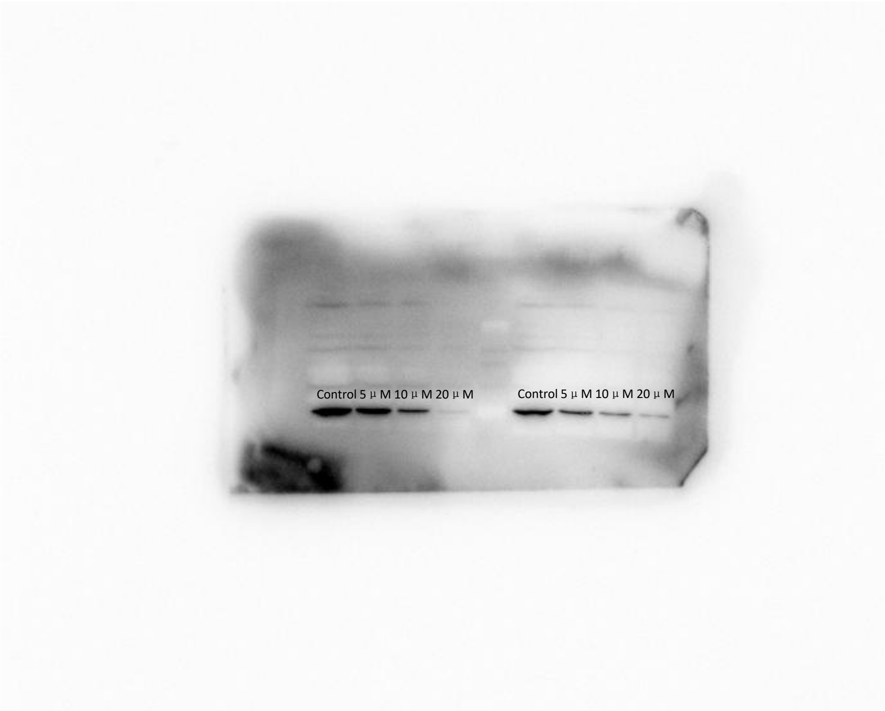

HIF-1a

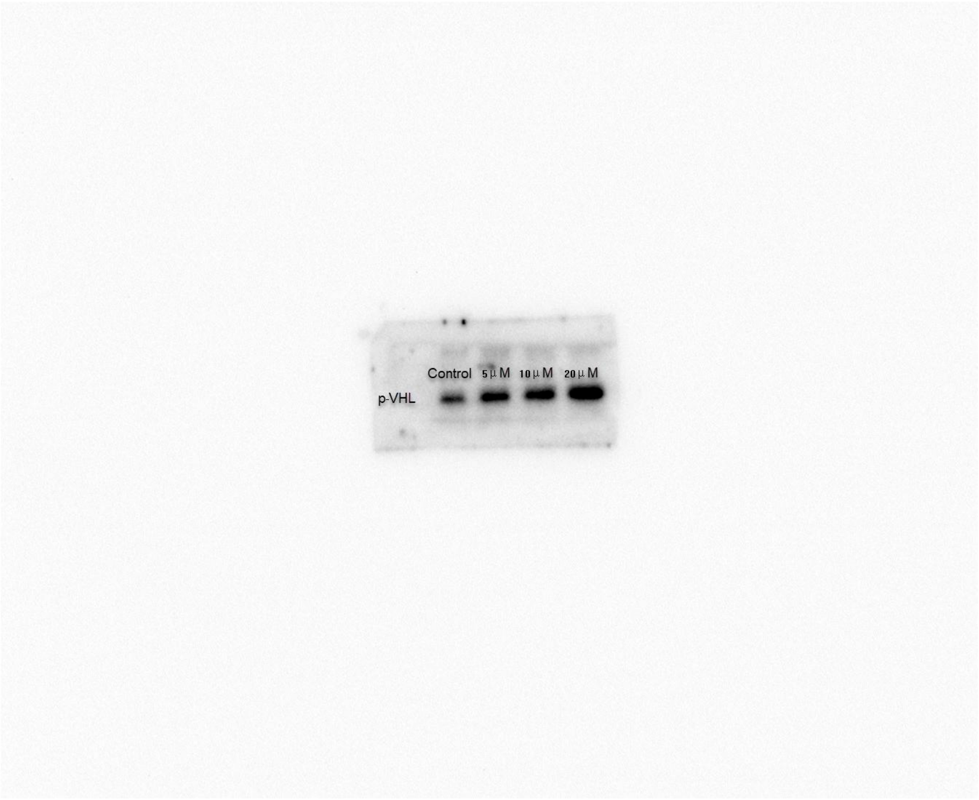
Control
5 μ M
10 μ M
20 μ M
p-VHL

P-VHL

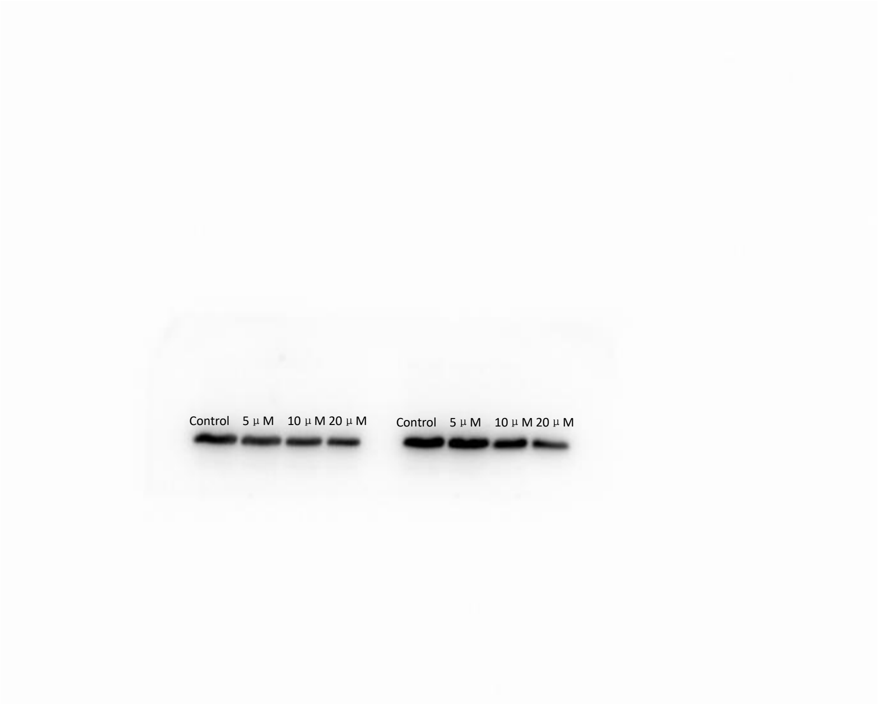
Control
5 μ M
10 μ M
20 μ M
Control
5 μ M
10 μ M
20 μ M

β -actin

Figure 5A

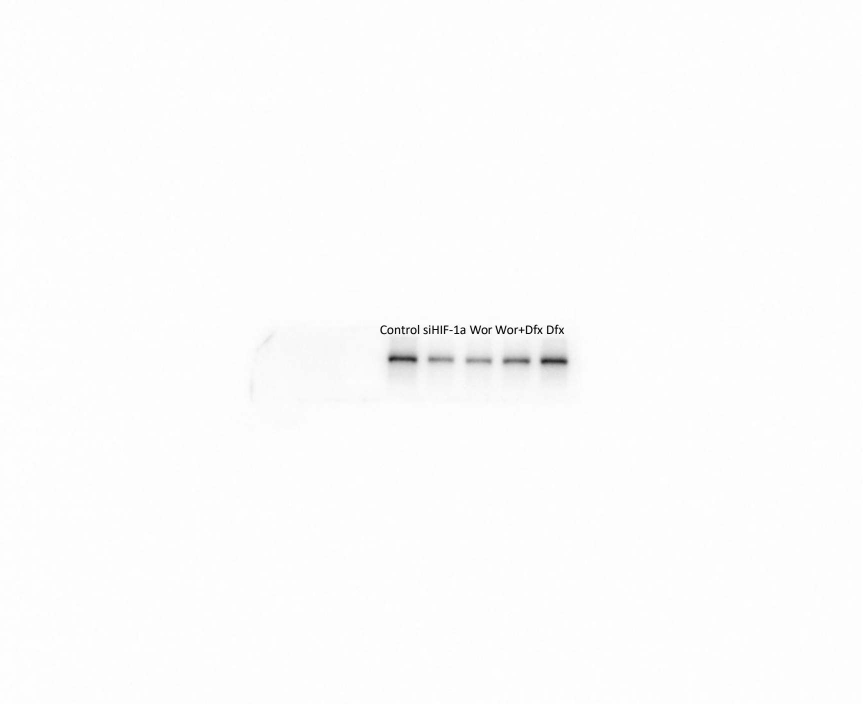

HIF-1a

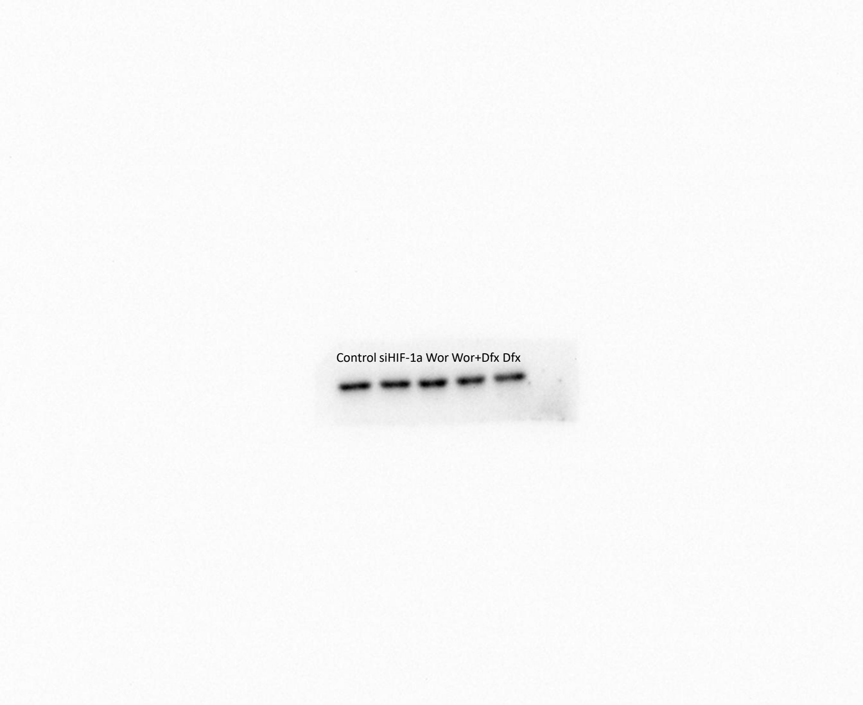

β -actin

Figure 5C

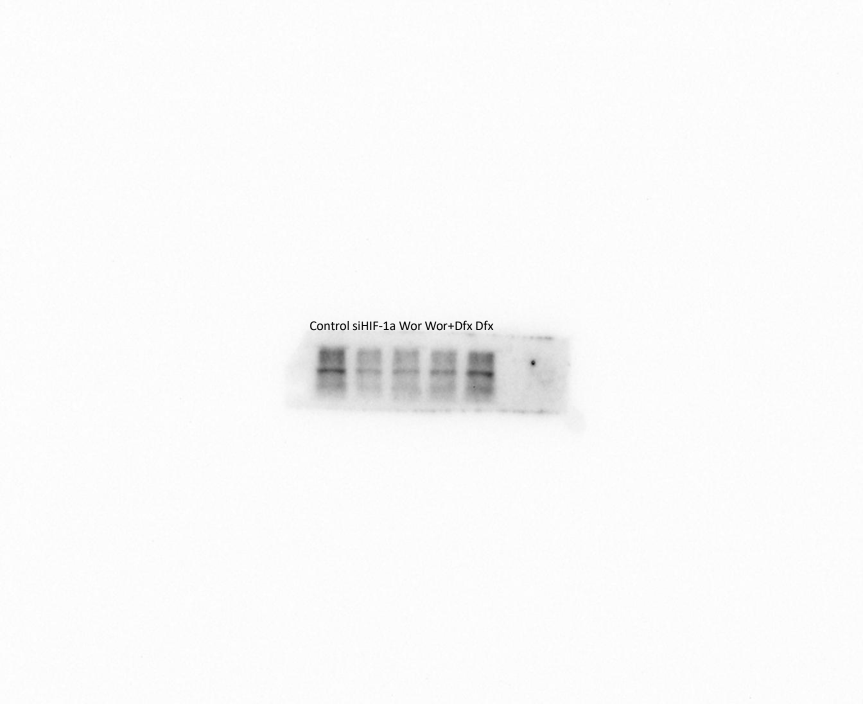

HK-2

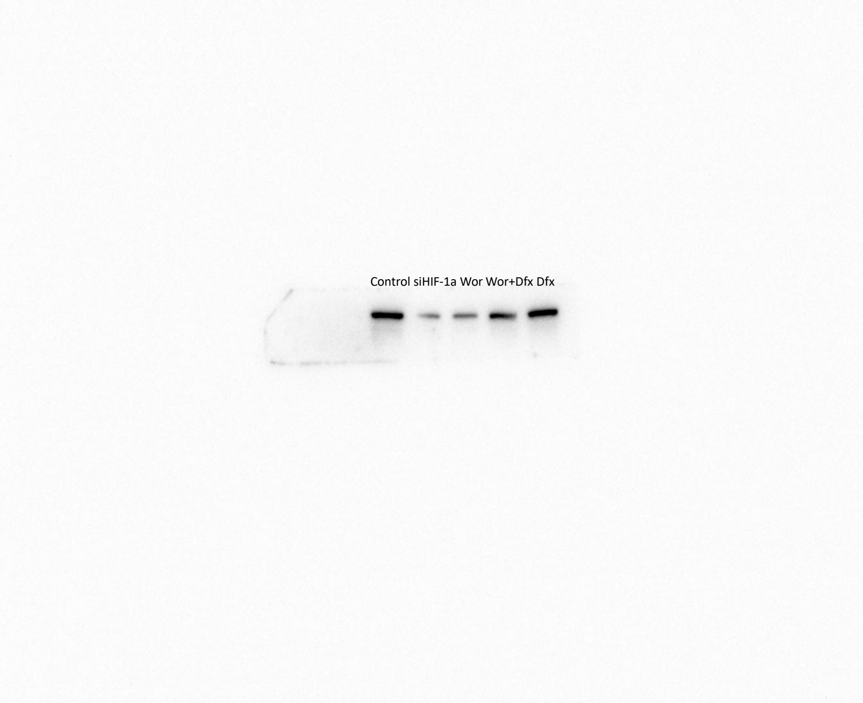

PKM-2

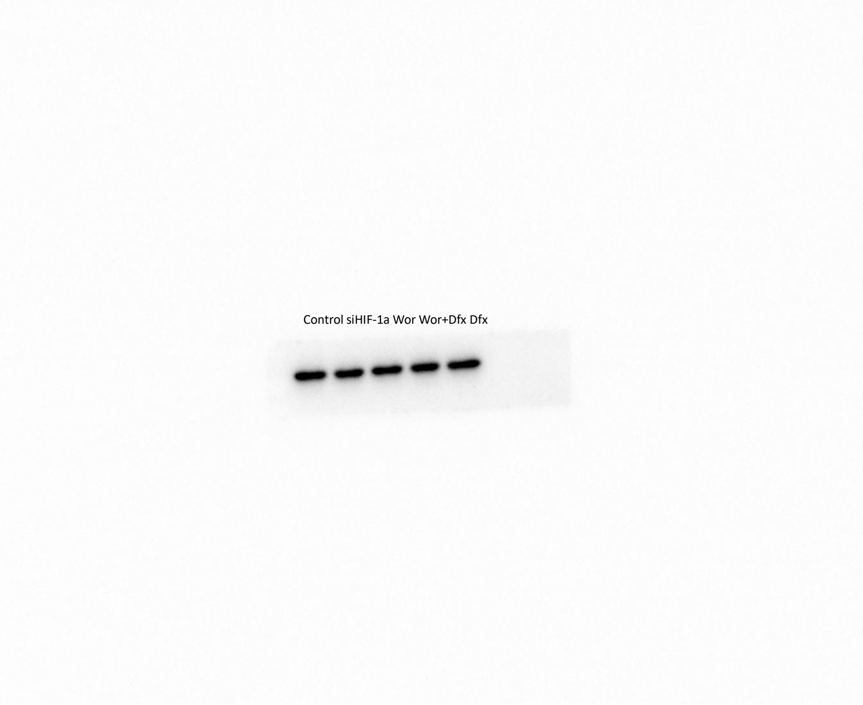

β -actin
